# Correlation of Blood Oxidative Stress Parameters to Indoor Radiofrequency Radiation: A Cross Sectional Study in Jordan

**DOI:** 10.3390/ijerph17134673

**Published:** 2020-06-29

**Authors:** Yazan Akkam, Ahmed A. Al-Taani, Salam Ayasreh, Abeer Almutairi, Nosaibah Akkam

**Affiliations:** 1 Department of Medicinal Chemistry and Pharmacognosy, Faculty of Pharmacy, Yarmouk University, Shafiq Irshidat st Irbid 21163, Jordan; nosaibahakkam@yahoo.com; 2 Department of Earth and Environmental Sciences, Faculty of Science, Yarmouk University, Shafiq Irshidat st Irbid 21163, Jordan; taaniun@yu.edu.jo (A.A.A.-T.); s.ayasreh@yahoo.com (S.A.); 3 Department of Life and Environmental Sciences, College of Natural & Health Sciences, Zayed University, Abu Dhabi P.O. Box 144534, UAE; 4 Science Department, College of Basic Education, Public Authority for Applied Education and Training, (PAAET), Alardyia P.O. Box 23167, Safat, Kuwait; am.almutairi@paaet.edu.kw

**Keywords:** electromagnetic radiation (EMR), glutathione S transferase (GST), power density, cell phone towers, oxidative stress

## Abstract

*Background*: Electromagnetic pollution is a general health concern worldwide, as cell phone towers are ubiquitous and are located adjacent to or on the roof of schools, and hospitals. However, the health risks are still inconclusive. This cross-sectional study evaluated the potential effect of electromagnetic radiation generated from various resources including cell phone towers on blood glutathione S transferase activity (e-GST) and total antioxidant activity of the Jordanian population. *Methods*: The power density of three districts in the city of Irbid, Jordan was mapped to generate “outside the houses” and “inside the houses” maps. The effect of categorical variables (gender, using a cell phone, presence of Wi-Fi modem, previous exposure to medical imaging) and continuous variables (distance from the base station, the elevation of the house, the duration of stay in the house, power density outside houses, power density inside houses) on e-GST and total antioxidant activity were investigated. *Results*: The EMR generated outside the houses—including cell phone towers—did not reach inside the houses at the same power and had no significant influence on e-GST activity. The EMR inside the house, which primarily came from internal resources, has a significant effect on e-GST activity. The duration of stay inside the house, the use of cell phones, and the presence of a Wi-Fi modem had a proportional effect on e-GST activity. The total antioxidant activity was statistically equal between the tested and control groups. **Conclusions**: Several factors such as building materials restricted the penetration of EMR reaching inside the houses. EMR generated inside rather than outside the houses had a proportional effect on e-GST. The differences in e-GST were compensated successfully by other antioxidant mechanisms. Further research is needed to identify other possible sources of antioxidants, and to evaluate long-term effects and genetic polymorphism.

## 1. Background

The exceptional spreading of telecommunication in the 21st century has exposed humans to high levels of electromagnetic radiation (EMR) [1]. Subsequently, a new type of pollution, referred to as electromagnetic pollution has been generated. EMR can be classified into two types depending on the energy and frequency. The first is ionizing radiation (IR) and the second is non-ionizing radiation (NIR), which includes telecommunication and data transfer antennas. [2]. According to the European Commission, the sources of NIR can be sorted into four fields; radio frequency (radio, television, smartphones, tablets and microwave ovens), intermediate frequency (video screens, antitheft devices, card readers, metal detectors), and extremely low frequency (power transmission lines, home wiring), and static (natural) [3]. Therefore, nowadays, a person is constantly exposed to EMR emitted by different sources.

Mobile phones are one of the fastest-growing technologies, and to keep up with the high demand, cell phone towers have become ubiquitous [4]. However, the health risks of EMR emitted from cell-towers and base stations are still inconclusive [5]. Thus, concerns about potential public health have been raised worldwide [6].

It has been reported that non-thermal effects of EMR could induce lipid peroxidation in human erythrocytes [7], oxidative damage in rabbits and rat’s brains [8,9,10], and alteration of epidermal homeostasis [11]. EMR exposure may also induce changes in the endocrine system [12,13]. Furthermore, it has been suggested that the EMR generated by mobile phones might be harmful to reproductive function [14,15,16,17], induced conformational changes and misfolding in proteins [18,19], activated heat shock proteins [20,21], and induced oxidative stress (ROS) [22,23,24].

Among all potential effects of EMR, the ability to induce oxidative stress drew attention. Oxidative stress is a process linked to many chronic diseases [22,23,24]. Glutathione S-transferases (GST) are enzymes that protect organisms from oxidative stress, including endogenous and exogenous compounds [25,26]. Erythrocyte GST (e-GST) represent a sensitive biomarker that is overexpressed in the case of increased blood toxicity [27,28]. Hence, they may serve as biological markers to detect oxidative stress [29].

Jordan lacks such studies to investigate the potential impacts of EMR on the population. This cross-sectional study mapped the power density generated from cell phone base stations and then compared the values “outside the houses” to the “inside the houses” in a rural area (Hofa, Habaka and Juhfieh). Uniquely, this study was intended for the general healthy population, so volunteers were reached at their houses. Later, the e-GST activity and total antioxidant activity were measured. The effect of categorical variables (gender, using a cell phone, presence of Wi-Fi modem, previous exposure to medical imaging) and continuous variables (distance from the base station, the elevation of the house, the duration of stay in the house, power density outside houses, power density inside houses) on e-GST was investigated.

Electromagnetic pollution is a general public health concern in Jordan, as there are many cell phone towers located adjacent to or on the roof of schools, residential buildings and hospitals. It has been reported that base stations in Jordan are arbitrarily installed all over the place form different cell phone companies [5]. Furthermore, the Electromagnetic frequency (EMF) transmitted from base stations in Jordan reached high levels and there were significant disparities between national radiation exposure limits and international guidelines [5]. Hence, a recommendation was directed to decision-makers in Jordan to take serious steps toward reducing radiation exposure, thus reducing health risks as much as possible. Moreover, the researcher emphasized that cell towers near schools, hospitals and residential areas must be moved [5].

## 2. Methods

### 2.1. Institutional Review Board (IRB) Approval

The study was conducted per the international ethical standards outlined by the Declaration of Helsinki [30]. Voluntary participation was applied, and participants were informed of their freedom to withdraw from the study at any time. Informed written consent including purpose, nature, and potential risks was obtained from all subjects. Subsequently, no samples were obtained from any subject younger than 18 years old. Information regarding individual characteristics (age and gender), health status (chronic diseases, medications in general and particularly antihistamine and paracetamol) and other related factors, including duration of stay at home per day, presence of Wi-Fi router, the usage of a cell phone, and exposure to any diagnostic EMR such as X-rays, were collected by the questionnaire

### 2.2. Study Design and Participants

**Figure 1:**
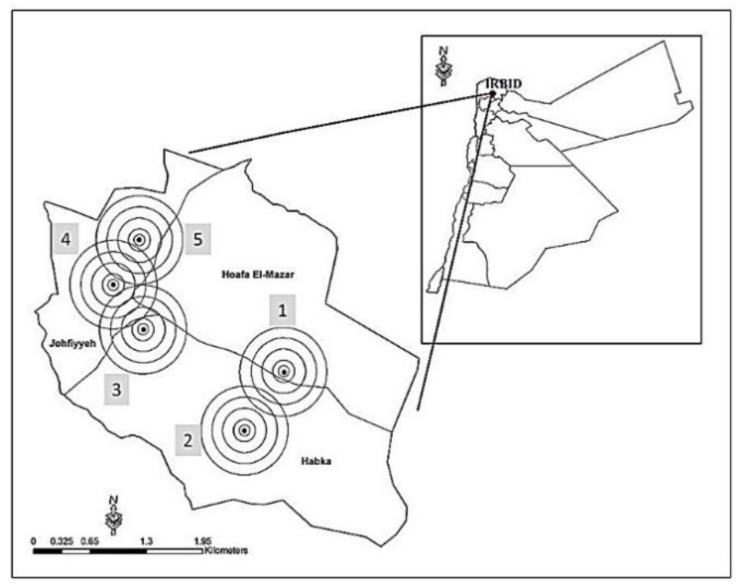
The geographical location. The area of the study is located in northern Jordan (city of Irbid). The amplified map showing the distribution of cellular stations with the tested (Hofa, Johfiah, and Habaka). Cell phone towers assigned by Arabic number (1–5).

All participants were living in a village in the city of Irbid in the north of Jordan. The selected area was divided into three districts: Hoffa, Johfiah, and Habaka, where approximately 13,000 citizens live in an 8 km^2^ area. Moreover, this area enclosed 5 cellular base stations operated by three different cellular companies, as shown in Figure 1. Each tower was identified by a numerical Arabic number (1–5).

The EMR mapping was carried out as described previously [5,31,32] using radio frequency (EMF) strength meter (Extech Instruments frequency range 50 MHz to 3.5 GHz Model: 480836/USA). The RF-meter was calibrated, and linearity and frequency responses were checked before use. The measurement was carried in the period of August to December/2017.

**Figure 2:**
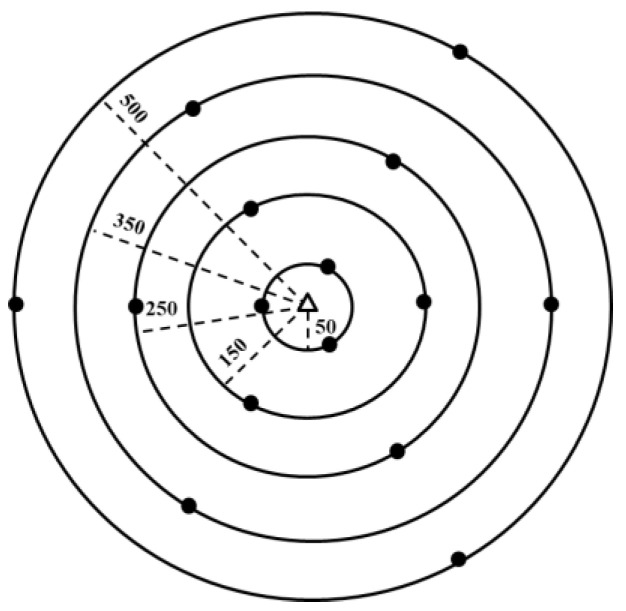
Experimental design for mapping the electromagnetic field around the cellular station. (●) Assigned points for measuring the power density. (∆) The cellular station. Numbers are distance in meters.

Five virtual circles around each cellular station were allocated with different radii: 50, 150, 250, 350, and 500 m (Figure 2). In each circle, 3 points were assigned, so a total of 15 points were assigned around each tower. The geographic information system (GIS) software was then utilized to assign the exact location coordinates (latitude, longitude, and altitude) and to ensure the even distribution of the points around the circumference. Later, the exact location of the imaginary spots was identified using a geographic position system (GPS).

A certified telecommunications network engineer measured the electric field (V/m), magnetic field (mA/m), and power density (µW/cm^2^). The power density data were then processed in a digital map and interpolated using geographic information system software (ArcGIS version 10.3 software, ESRI, Redlands, CA, USA). ArcGIS was used to construct a 2D map and ArcScene to draw the 3D map. In the process of assigning locations around the towers, a couple of points were taken into consideration: the visibility (accessibility) of the cellular tower and avoiding the high voltage cables. For “outside house” measurements, the elevation of the meter from the ground and the planarity were kept constant. Each measurement was acquired over a minimum period of 15 s. The maximum value after stabilization was recorded, averaged, and then expressed as the mean ± SD.

For indoor EMR, the power density was measured in bedrooms right before the blood sampling, as described previously [32].The RF-meter was about 1 m from the walls and 1.2 m above the ground—and moved around a circle of 0.25 m radius. The meter was orientated in different directions to obtain the maximum strength above the bed. The power density was measured over a 6 min time frame according to the International Commission on Non-Ionizing Radiation Protection (ICNIRP) guidelines from 1998 [33] and the updated version from 2018 [34]. Each reading was recorded in triplicate on two separate days (total of 6 readings for each point). Moreover, the power densities were re-measured inside and outside the houses during the sample collection. Subsequently, two different digital maps representing the RF radiation were constructed and entitled “inside house” and “outside house”.

### 2.3. Blood Samples Collection

Healthy participants between 18 and 45 years old provided blood samples (n = 132). Volunteers with a history of uncontrolled systemic hypertension, hyperlipidemia, cardiac failure, hepatic failure, kidney disease, diabetes, cerebrovascular diseases, peripheral vascular diseases or coagulopathy, as well as smokers, were excluded. The control group was defined as people living in an area where the power density inside and outside the house was zero (n = 20). A trained nurse drew blood from the left median cubital vein of the participants. A 4 mL blood sample was taken from each person using a vacuum tube with EDTA-K 2 (Ruiqi Technology Ltd, Chengdu, Sichuan, China.). The tubes were placed immediately in dry ice, then stored at −80 °C and analyzed within a week after storage.

### 2.4. GSTs Activity Measurements

The activity of GSTs in total blood was determined spectrophotometrically with the microplate reader (Bio-Tek ELx800, Winooski, VT, USA), as described previously [35]. Briefly, blood samples were hemolyzed in 50 volumes of ice-cold water. Hemolyzed samples were incubated with 0.5 mM GSH and 1 mM of 1-chloro-2, 4-dinitrobenzene in 0.197 mL of 98.5 mM potassium-phosphate buffer, pH 6.5. The enzymatic activity assay was performed at (37 °C) with λ = 340 nm in a 96-well plate (Catalog Number 655101). The absorbance at 340 nm was measured after a 1 min lag time and every minute over 10 min. Each assay was measured in triplicate.

Total protein concentration was measured using the bicinchoninic acid protein assay kit (Sigma-Aldrich, Sigma-Aldrich, Steinheim, Germany). GST activity was expressed as enzyme units (U) per gram of Hb (U/gHb): one unit represents the amount of enzyme that catalyzes the conjugation of 1 µM of GSH to CDNB in 1 min at 37 °C. Importantly, the activity of GSTs was linearly related to its expression, hence no coefficient of determination (R^2^) less than 0.97 was accepted [12].

### 2.5. Total Antioxidant

The potassium permanganate method was performed as described by Zhang et al. [36]. Briefly, the hemolyzed blood was diluted with distilled water into a series of concentrations (1:10; 1:20; 1:40; 1:80; and 1:160). For each concentration of blood sample, a 20 L aliquot was added to a 96-well ELISA plate. A blank control (no blood) was established. Then, 100 L of 5 mmol/L of KMnO4 solution was added to each well and mixed uniformly by shaking. The mixture was placed in a water bath at 37 °C for 30 min; after that, the absorbance (OD) was measured at λ = 570 nm. The total antioxidant of the blood (Ta) was calculated as described previously [36]. The measurements were performed in a point-by-point manner, where the change in the OD values is correlated to the oxidizing agents reacting with reducing blood samples. The following equation was used:

(1) $$Ta=\frac{100}{\left[OD1+2\times \left(OD2+OD3+OD4\right)+OD5\right]}$$

### 2.6. Statistical Analysis

Statistical analysis was performed using the statistical package for social sciences (SPSS-16, Chicago, IL, USA). The e-GST results were expressed as mean ± standard deviation (SD). A *p* value < 0.05 was considered statistically significant. Student’s *t*-test was used for the continuous variables, while two independent-sample *t*-test was conducted to evaluate the categorical variables. One-way analysis of variance (ANOVA) was used to compare mean values in all groups, followed by multiple comparison post hoc tests. The logistic regression model was also used to assess the associations with and without adjustment for potential confounding factors such as gender. The independent-sample *t*-test was used to assess the different means of the antioxidant capacity in serum between samples and the control.

## 3. Results

### 3.1. Characteristics of the Study Area

**Figure 3:**
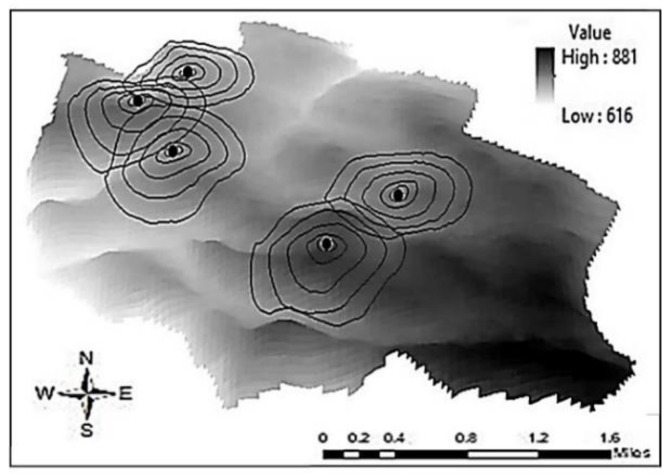
Contour map (elevation surface) of the tested area using geographic information system (GIS) software. The gradient gray color represents the elevation (in meter) from the sea level. The dark circles are the cell phone towers.

The tested area is relatively flat with minimal terrain, as seen in the topographic map (Figure 3). Additionally, the area does not have any natural physical barriers, as well as any buildings with more than two stories high (6–7 m). The descriptions and the characteristics of each cell phone tower are summarized in Table 1. The frequencies produced by cell phone towers measured in this research were between 880 MHz and 2.8 GHz (Table 2).

### 3.2. Power Density Maps

**Figure 4:**
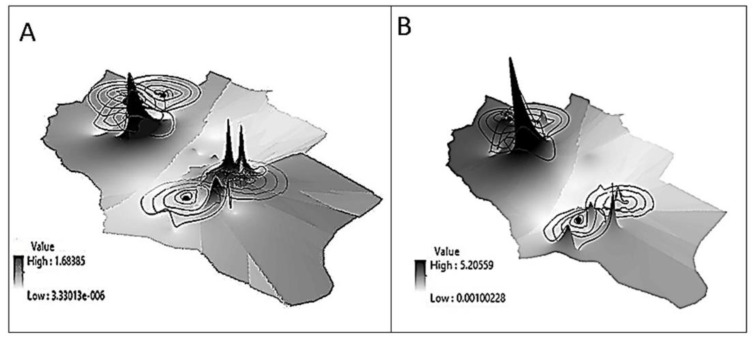
Three-dimensional maps of the tested area showing the power density (μW/cm^2^). With a color gradient. (**A**) Outside the houses. (**B**) Inside the houses. The color gradient showing the average power density.

After the correlation of power density data with the geographical information in ARC-GIS software, a 3D map of the power density was generated for the reading outside and inside the houses (Figure 4). From the power density data measured “outside the houses”, the values across the area were not evenly distributed. The average power density ranged between 3.3 × 10^−6^ and 1.68 µW/cm^2^ (Figure 4A). The same observation was noticed in the “inside the houses” map (Figure 4B), where the distribution of power density was uneven. The average power density ranged between 0.0001 and 5.2 µW/cm^2^.

### 3.3. Sample Selection and GST Activity

**Figure 5:**
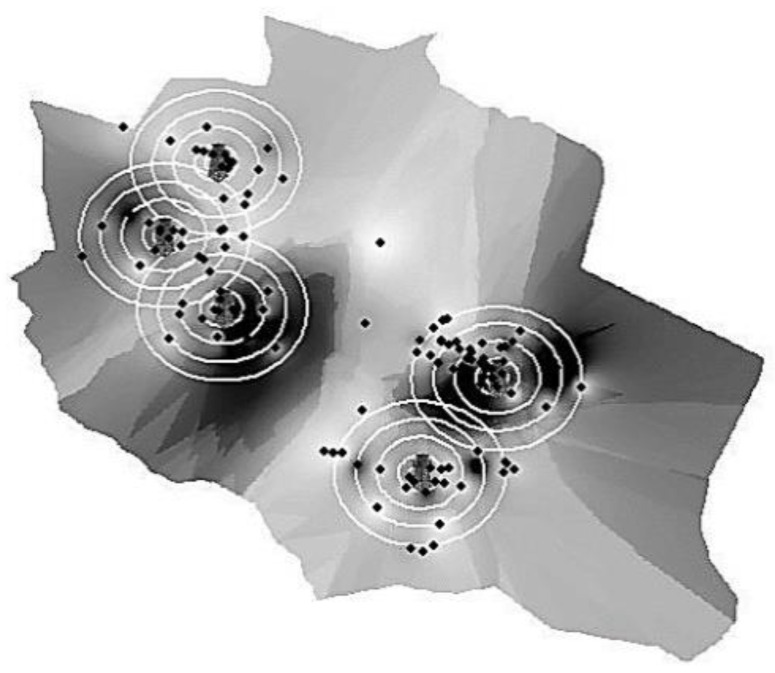
Distribution of volunteers’ houses on the power density map.

**Figure 6:**
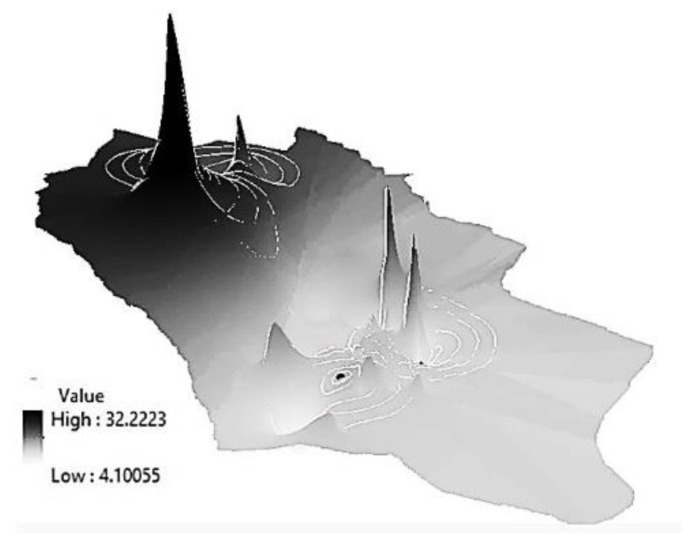
Three-dimensional map represents the glutathione S transferase (GST) activity.

One hundred and thirty-two people fitting into the selection criteria volunteered in this study over a 6 month period. Control participants were living in locations with no cellular coverage but within the same region. The control samples were assigned after the power density was measured in all regions and the 3D maps constructed (Figure 5). A 3D representation of the average GST activity is illustrated in Figure 6, with a maximum activity reaching 32 U/g/Hb, while the lowest was 4 U/g/Hb. The average GST activity within the control group was 10.5 ± 0.71 U/g/Hb.

### 3.4. Statistical Analysis

Baseline characteristics were presented per patient as percentages for categorical variables (Table 3). Categorical variables that have been correlated to the EMF were collected via the questioner, including has the volunteer previously been exposed to any medical imaging (X-ray, MRI, CT scan, etc.), does the volunteer use a cell phone, and does the volunteer have a Wi-Fi modem at the house?

To determine the possible influence of the categorical variables of the GST activity, two independent-sample *t*-tests were used (Table 3). As illustrated in Table 3, there are no statistically significant differences at the level of (*α* < 0.05) in the total GST activity attributed to gender or exposure to medical imaging. However, a significant difference in the GST activity was observed using both cell phones and Wi-Fi modems in houses.

The continuous variables that were analyzed using simple regression analysis were; the power density outside the house, power density inside the house, the elevation of the house from the sea level, and the horizontal distance between the house and the tower (Table 4). Furthermore, “daily exposure time” was assessed by a relevant question; “How many hours per day on average do you stay at the house?” (Table 4). 

As illustrated in Table 4, there was no statistically significant difference in the GST activity related to the following variables; power density outside the house, the elevation of the house, and distance from the tower at a significance level of (*α* < 0.05). This result was also confirmed by estimating the values of (B), (*β*), and (t) (Table 4). The value of (B) for tested variables was not statistically significant at (*p* < 0.05). Furthermore, the (t) calculated was higher than of the (t) tabulated. It is worth mentioning that “the house elevation” does have a significant relationship with GST activity at (*α* < 0.1). The positive sign of *β* indicates that tested variables have a positive relationship with the GST activity.

On the other hand, the variables “power density inside the house” and “duration of exposure” have a significant influence on the GST activity (Table 4). The statistical significance level was less than (*α* < 0.05) for both variables. The value (F) for “power density inside the house” and “duration of exposure” was 63.9 and 29.9, respectively. Although the R-value is low (0.202), “the duration of exposure” showed a significant (*p* < 0.05) impact on GST activity, with a standard coefficient *β* value of 0.45. According to *β* values, the relationship with GST activity was proportional.

To study the effect of all variables on GST activity, multiple regression analysis was conducted. Table 5 shows the regression analysis with the dependent variable “GST” and the five factors, namely, power density outside the house, power density inside the house, duration of exposure, the elevation of the house, distance from the tower, as independent variables. The ANOVA test was significant (*α* < 0.05), with an F-value of 18.993. Only power density inside the house and duration of exposure had a significant (*α* < 0.05) impact on GST activity, with standard coefficient *β* values of 0.503 and 0.351, respectively. The simple correlation coefficient (R) was 0.676, and the R^2^ was 0.457, which indicates that the “independent variables” explain only 46% of the relationship with the dependent variable (GST activity) and the remainder was due to other factors.

### 3.5. Antioxidant vs. GST Activity

**Figure 7:**
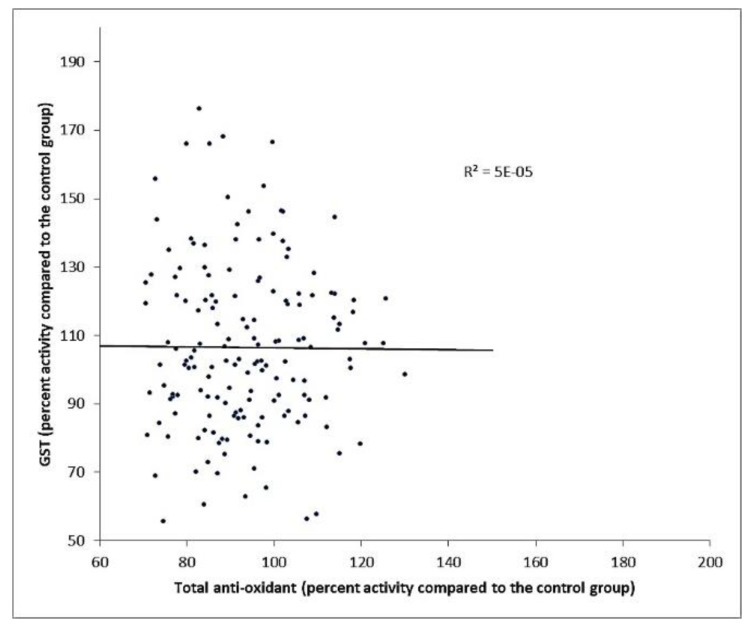
Linear regression between GST activity and total antioxidant activity.

The average antioxidant capacity in serum (Ta) for samples was very close to the control. The Ta value was 7.702 ± 0.33 and 7.964 ± 0.76 for the control and samples respectively. No significant differences between the two-population means were observed (Figure 7).

## 4. Discussion

In the current century, the world is overwhelmed by wireless telecommunication and electrical devices, especially with the introduction of social media. Consequently, a new type of pollution has been generated—referred to as “electromagnetic pollution”—where wireless and radio communication devices and some home appliances are significant contributors [37]. Despite establishing several theories, the effect of this pollution on human health is still an open question as there is no clear and definitive evidence of its negative influence [37].

EMR is a possible inducer of oxidative stress, and consequently increases the production of free radicals, which may lead to DNA damage [18,38]. Moreover, the induction of oxidative stress may not only occur through the production of reactive oxygen species but also via the weakening of the antioxidant mechanisms [39,40]. Subsequently, oxidative stress byproducts may serve as initiators and inducers of chronic diseases [40,41,42].

GSTs, as members of the antioxidant system, are important detoxification enzymes [43] and are responsible for the high-capacity inactivation of electrophilic compounds [44]. In general, an increase in GST enzymatic activity is due to the exposure to electrophiles or oxidative stress [45]. Among GST classes, blood GSTs were the main emphasis, as they are subjected to a high rate of internal reactive oxygen species produced from hemoglobin auto-oxidation [46]. Blood is the first compartment exposed to oxidative stress, including red blood cells. Furthermore, erythrocytes are subjected to high risk of oxidative damages due to the exposure to high oxygen tension, and lacking damage repair mechanisms [25]. Consequently, erythrocytes are entirely reliant on the antioxidant defensive components throughout their life span [47]. So, the induction of oxidative stress can be monitored or measured via studying the blood GST which includes serum GST and erythrocyte GST.

Most previous studies which investigated the effect of EMR on human health were based either on randomized trials applying controlled exposure conditions in a laboratory [44] or correlating occupational exposure to specific pathogenicity or physiological conditions [31,32,48,49,50]. In the former case, the data were scarce and the evidence for long-term effects was limited. In the latter case, there were no biological sample collections, nor exact measurement for the EMF.

### 4.1. Radiation and Study Design

Since there is more than one source of electromagnetic radiation with different frequencies (including cellphone EMR), the logic measurement would be the use of power density (rate of energy transferred per unit area) [51]. Power density is a cross product of the electric field strength and the magnetic field strength [5]. Generally, physical barriers may interfere with EMR via decreasing the signal strength and amplitude of the power [52,53]. The research study area was flat with limited topography, meaning it was less likely to interfere with the radiation signals. Furthermore, tested area did not comprise any physical barriers such as tall buildings, which allows the signal to reach long distances.

The base station is designed to enable mobile phones to transmit and receive enough signals for communication up to a few kilometers. It has been stated that the distribution of the radiation from the antenna depends on the tower height and downward angle, therefore, the maximum power at ground level is reached 50–300 m from the tower [54]. Hence, in the experimental design, the power density was measured at distances between 50 and 500 m away from the tower. Moreover, the height of the building might be related directly to the amount of power that reached the house. Thus, the elevation of the house was taken into consideration. As seen in Table 4, the elevation in the current study did not show a significant influence on the GST activity at (*α* < 0.05), but it did at *α* < 0.1, with a proportional relationship (the activity increases as the house elevation increases).

The amount of power density produced by cell phones relies on the number of telecommunication structures found in the area, the cell phone network traffic, and the distance of cell phones from base stations [55]. Hence the distance from the cell phone towers is a legitimate variable. Under the tested conditions, the variable “the distance” did not possess a significant effect on GST activity at *α* < 0.05. It has been suggested that the sample size in multiple linear regression analysis is 20 subjects per each predictor [56]. Since there were five continuous variables in this study, a sample size of 100 would be representative. However, a sample size of 132 was used to have more generalizable results.

The percentage of females in the study was 56%, while the proportion of males was 44%. The proportion of females in the sample was slightly higher than males. The distribution of the sample was close to the distribution of the population by gender. The total population in the study area was 13,218 people, of which 52% were males (6898) and 48% were females (6320) (Department of Statistics, 2018). Moreover, no statistically significant variation in e-GST activities according to gender was measured (Table 3).

According to the results, the power density outside the houses did not show a significant correlation (*α* > 0.05) with the GST activity nor with the power density inside the house. This implies that EMR produced from the cell phone towers was weakened before reaching inside the houses at the same power.

### 4.2. Power Density Outside vs. Inside

The notable observation regarding the power density map is the values outside the houses did not follow the same pattern as the reading inside the house (Figure 4). Generally, buildings constructed with metal frames are bad for cell phone signals reception. Metals such as aluminum, copper, and steel reflect electromagnetic waves instead of letting them pass through [57]. Several building materials can affect the cellular signal, such as the brick, the thickness of the brick wall, concrete, plaster, and even the mortar between the bricks. Glass windows can also reflect the signal away from the house [58]. In Jordan, steel bars are commonly used in buildings, and house roofs are generally composed of steel and cement. Moreover, most houses have steel security bars on their windows as a general practice to improve safety.

Rebar steel bars in the foundation, frame and roof, along with the security bars, induce an effect akin to a Faraday cage, which may reduce the signal from outside. It has been reported that the power level inside a building can be up to 100 times lower than that outside the building depending on the number of windows and the structure of the walls [51]. Tree and foliage can also affect the signal, and are even capable of blocking the signal [59]. The degree of blockage is varied according to the age and the size of the trees [58]. The studied area was rural. Hence, most of the volunteers were living in separate houses with gardens mainly planted with evergreen olive oil trees.

It worth mentioning, as seen in Figure 4, that there was a location where the power density inside and outside was extremely high (near tower 4). This phenomenon might be because the house was at the same height as the base station and the antenna was oriented toward the house window. Moreover, the windows did not have any mesh security wire.

### 4.3. Power Density Inside vs. GST

The power density inside the houses was measured in bedrooms, as it is the place where the participants spend most of their time. Power density inside houses depends on both outdoor mobile phone antennas and indoor sources, such as mobile phones, wireless communications applications and other electric appliances, such as microwaves [51,60]. In the tested area, several sources of radiation “inside the house” exist such as cell phones and Wi-Fi modem. Moreover, none of the selected houses enclosed any wireless DECT phone base or handsets.

What does not allow the signal to get inside, will not allow the signal generated inside to leave outside. Therefore, the power density may be built up from the in-house resources. Moreover, it has been stated that frequencies from 10 MHz to 300 GHz can be carcinogenic, which includes some household appliances (such as microwaves and telecommunications devices) [61]. There is a correlation between radiofrequency and body penetration. When the frequency increases, it penetrates less into the body tissues. Therefore, the penetration of Wi-Fi frequencies is lower than that of cell phone frequencies [62]. Another difference between different sources of EMR is the operation distances. In general, cell phones operate at maximum power and are held next to the body, while the Wi-Fi modem is located at a distance from the human body. As it is known, the power density decreases in proportion to 1/d^2^ (where d is the distance to the source) [61]. Hence, people are exposed to EMR when talking with cell phones, whereas EMR emitted from Wi-Fi affects the entire body. It is important to mention that other determinant factors are also involved in the interaction between EMR and the human body such as frequency, permeability and dielectric constant [63].

The majority of digital cell phone radiation employs extremely low frequencies (ELF) necessary for the modulation and for increasing the capacity of transmitted information by pulsing the signal. Interestingly, the use of ELF pulsing frequencies has been found to increase the bioactivity [64]. It has been shown that pulsed radiation (cell phones) is more bioactive than constant radiation (Wi-Fi modem) [62]. It is thus a legitimate finding that the existence of a cell phone “inside the house” affect the GST level. Largely, the term “using cell phones” is a general concept, as the intensity of radiation produced by cell phones varies significantly each moment during a usual phone-conversation. The intensity depends on signal reception, the number of subscribers sharing the frequency band at each moment, air conductivity, and “speaking” versus “nonspeaking” mode [65]. Hence, the biological effect of cell phones can vary between users. The more the amount of carried information is increased (by adding text, speech, pictures, music, video, internet, etc.), the more unpredictably varying the cell phone signals become [65].

It has been reported that continuous exposure to Wi-Fi radiation induces oxidative stress in rats and increases the GST activity [65]. This effect is also confirmed by these results, as the presence of a Wi-Fi modem had a significant effect on GST activity. This finding also fits well with the effect of power density inside the house. This may explain why the “time of exposure” has a proportional relationship with the GST activity. The greater the exposure to in-house high EMR, the higher the GST activity. Moreover, it is thought that public exposure levels arising from Wi-Fi are lower than those from mobile phones [66]. It is worth mentioning that using an RF-meter alone cannot determine the source of radiation that generates the most oxidative stress.

Exposure to medical diagnostic imaging (X-ray, CT-scan, MRI) is a cause of oxidative stress [67], which may interfere with the GST activity. Hence, this confounding variable was evaluated. According to the results (Table 3), previous exposure to medical imaging did not imply a statistically significant effect on the GST activity.

### 4.4. Total Antioxidant vs. GST Activity

GST is not the only enzyme to be induced to counteract ROS formation. A series of antioxidants and detoxification enzymes can be initiated, such as superoxide dismutase, catalase, glutathione peroxidase, thioredoxin, peroxiredoxin [68]. So, the antioxidant activity in blood is not limited to GST. Humans have a highly complex antioxidant protection system with multiple components; endogenous antioxidants (such as bilirubin, lipoic acid, ubiquinone), dietary antioxidants (such as vitamin E, vitamin C, Beta carotene, flavonoids) and metal-binding proteins (albumin, ferritin, transferrin) [69]. So, the logic is to evaluate the potential influence of GST activity on the blood total antioxidant.

Despite the variation in the GST activity, only minor changes were observed in the total antioxidant activity. As seen in Figure 7, the variation in total antioxidants was statistically ignored; there was no linear relationship between the total antioxidant and the GST activity as R^2^ = 1^−5^. Hence, GST activity explains none of the total antioxidant variations around its mean, and any relationship was coincidental. This implies that the human body successfully compensates for the changes in the GST activity.

## 5. Conclusions

This study was not restricted to mobile phone base stations’ frequency bands; it also assessed the exposure to other sources of radiofrequency electromagnetic fields in daily life, such as mobile and cordless phones and common home appliances. Under the testing conditions, the EMR from cell phone base stations most likely did not reach inside the houses at the same power. Factors such as house building materials may reduce the signal strength inside the houses. Subsequently, the amount of power density outside the houses exhibited no effect on blood GST activity.

The results suggest that the EMR inside the house, which primarily came from internal resources, has a proportional effect on the blood GST activity. Therefore, the duration of stay inside the house and the presence of a Wi-Fi modem also had a proportional effect on e-GST activity. Moreover, the results show that the use of cell phones increased e-GST activity. Despite that, the statistics showed that “independent variables” explained only 46% of the relationship. This implies that other sources are interfering with the e-GST activity. The total antioxidant activity was statistically equal between the tested and control group. This implies other antioxidant mechanisms compensated for the variation in blood GST activity. Further research is needed to identify other possible sources of antioxidants.

## 6. Limitations and Future Work

The biggest obstacle in cross-sectional studies intended for a healthy population is the agreement to participate. In such a community, the lack of motivation has constrained participation. Despite the presence of a professional female nurse, the fear of getting an infection from an unsanitary needle dissuaded participation. Moreover, the concept of volunteering is hugely unpopular in the community, especially vein sampling. Furthermore, Jordanian mores, especially when half of the participants were females, limited volunteering.

GSTs and total antioxidant enzymes are highly ubiquitous and non-specific, as it can be affected by a large number of confounding variables. Therefore, this study tried to exclude as many confounding variables as possible in the selection criteria (questionnaire), but there is a possibility that other factors may interfere with GST and antioxidant activities, such as pollutants in the air, water and food, secondary smoker effect, occupational exposure, time spent living in the region, socioeconomic position and genetic composition.

In future work, detailed spectrum analysis should be used to identify the source and the frequency of power density at each point. Due to the development of new technology (5G-EMF frequencies > 6 GHz), ICNIRP changed the guidelines and added several regulations in 2020, such as the whole-body exposure restriction. Therefore, in future works, these changes should be taken into consideration.

The real digital mobile phone emissions change constantly and unpredictably; especially with recent phone generations (4G, 5G.). Hence, the peak measurement should be taken into consideration, as well as other variables that influence the cell phone emission such as the average use and the carried information). Finally, the absence of evidence of harm should not necessarily be interpreted as evidence that no harm exists. Further research should focus on long-term effects and should include children and adolescents

## 7. Availability of Data and Materials

The datasets used and/or analyzed during the current study are available from the corresponding author on reasonable request.

## Figures and Tables

**Table 1:** Basic information about cellular towers in the tested area. The assignment of the towers is in Figure 1.

Tower	Description	Height (m)	Tools	Microwave Link	Datalink	Companies
1	Green field	40	2G/3G	1	0	O/U
2	Roof top	26	2G/3G	1	0	Z
3	Roof top	21	2G/3G	6	7	U
4	Green field	30	2G/3G/4G	4	0	Z
5	Green field	30	2G/3G	2	0	U

**Table 2:** Cell phone frequencies of each company (from spectrummonitoring.com).

X	Technology	Cell Phone Frequencies (MHz)
Z	2 g	880–885/925–930/947.5–960
	3 g	1765–1785/1860–1880
	4 g	1945–1965/2135–2155
O	2 g	900–902.5/935–947.5
	3 g	1755–1765/1850–1860
	4 g	1935–1945/1965–1970/2125–2135/2155–2160/2550–2560/2670–2680
U	2 g/3 g	1730–1750/1825–1845
	4 g	1970–1980/2160–2170

**Table 3:** Demographic characteristics of study volunteers.

Characteristics	N = 132	*T*-Test				Interpretation
**Gender**		***t***	**Sig.**	**F**	**Sig. (2 tailed)**	
Male	58 (44%)	−0.265	0.827	0.048	0.806	No differences between the total GST activities in the two groups
Female	74 (56%)	−0.245			0.807	
**Using cellphone**						There are significant differences at the level of (α < 0.05)
Yes	37 (28%)	−5.652	0.000	13.260	0.000	
No	95 (72%)	−5.951			0.000	
**Wi-Fi modem at house**						There are significant differences at the level of (α < 0.05)
Yes	74 (56%)	−5.322	0.000	16.452	0.000	
No	58 (44%)	−4.952			0.000	
**Previously Exposed to Medical Imaging**						No differences between the total GST activities in the two groups
Yes	55 (42%)	1.430	0.144	2.158	0.155	
No	77 (58%)	1.568			0.121	

**Table 4:** Regression analysis for tested continuous variables and GST activity.

Variable	Mean ± SD	*P* Value	F	R^2^	B	β	t	Type of Relationship
Power density outside the house (μW/cm)	0.428 ± 0.2	0.447	0.58	0.005	0.654	0.07	0.764	NO relation
Power density inside the house (μW/cm)	0.789 ± 1.2	0.0001	63.8	0.589	0.784	0.589	7.99	proportional
duration of exposure (h)	17.72 ± 4.53	0.0004	29.9	0.202	0.39	0.45	5.469	proportional
elevation of the house (m)	625.2 ± 184.6	0.075	3.23	0.026	0.043	0.162	1.797	NO relation
distance from the tower (m)	384.18 ± 85.3	0.182	1.8	0.015	0.003	0.122	1.343	NO relation

**Table 5:** Regression analysis with dependent variable GST activity and the five factors, that is, power density outside the house, power density inside the house, duration of exposure, the elevation of the house, distance from the tower as independent variables. ^a^ Dependent variable: GST activity. **^b^** Predictors: (constant), power density outside the house, power density inside the house, duration of exposure, the elevation of the house, distance from the tower.

ANOVA ^a^							
Model	Sum of Squares	df	Mean Square	F	Sig.	R	R^2^
Regression	800.682	5	160.136	18.993	0.000 ^b^	0.676	0.457
Residual	952.735	127	8.431				
Total	1753.418	132					

**Model**	**Unstandardized Coefficients**		**Standardized Coefficients**		**Sig.**
	**B**	**Std. Error**	**Beta**	**t**		
(Constant)	3.718	1.203		3.091	0.003
Power density inside the house	0.715	0.102	0.503	6.987	0.000
duration of exposure	0.303	0.062	0.351	4.848	0.000
distance from the tower	0	0.002	−0.009	−0.097	0.923
elevation of the house	0.017	0.023	0.068	0.747	0.456
Power density outside the house	0.37	0.653	0.042	0.567	0.572

## Abbreviations

DECT	Digital Enhanced Cordless Telecommunications
e-GST	erythrocyte Glutathione S transferase
ELF	Extremely Low Frequencies
GSH	glutathione
EMR	electromagnetic radiation
IR	Ionizing Radiation
ICNIRP	International Commission on Non-Ionizing Radiation Protection
NIR	Non-Ionizing Radiation
GIS	geographic information system
GPS	geographic position system
RF	Radiofrequency
Hb	hemoglobin
